# DEVELOPMENT AND IMPLEMENTATION OF THE COGDRISK DEMENTIA RISK ASSESSMENT TOOL AND INTERACTIVE WEBSITE

**DOI:** 10.1093/geroni/igac059.2814

**Published:** 2022-12-20

**Authors:** Scherazad Kootar, Ruth Peters, Md Hamidul Huque, Ranmalee Eramudugolla, Kaarin Anstey

**Affiliations:** University of New South Wales, Sydney, New South Wales, Australia; University of New South Wales, Sydney, New South Wales, Australia; University of New South Wales, Sydney, New South Wales, Australia; University of New South Wales, Sydney, New South Wales, Australia; University of New South Wales, Sydney, New South Wales, Australia

## Abstract

We developed a comprehensive risk assessment tool for dementia – Cognitive Health and Dementia Risk Assessment (CogDrisk) and a version specifically for Alzheimer’s disease called CogDrisk-AD that could be applicable in low and high-resource settings. This tool incorporates risk and protective factors identified through systematic synthesis of observational studies that report risk ratios. Risk and protective factors included in the tool were selected on the strength of evidence as well as the availability of measures that are practicable in a range of clinical and research contexts. Seventeen risk/protective factors were identified for inclusion in the dementia algorithm to estimate the risk of dementia while sixteen factors were identified for the AD model, with an overlap in the majority of the factors. CogDrisk and the CogDrisk-AD were predictive of dementia and AD when validated across four high-quality international cohort studies. To enable the CogDrisk tool to be implemented in practice our team has developed an interactive website where individuals 18 years and above can complete the CogDrisk questionnaire, obtain a personalised risk profile, and receive feedback on their risk profile. The website was developed with the capacity to collect and store data. We anticipate that the tool can be used by members of the public, in clinical settings and as a screening or outcome measure for clinical trials.

